# Roles of Mitochondrial Fusion and Division in Harmine Derivative H-2-168-Induced Neurotoxicity

**DOI:** 10.1155/jimr/6678026

**Published:** 2025-06-02

**Authors:** Yuehong Gong, Meichi Pan, Hang Ren, Dongling Peng, Meiling Zhao, Yicong Zhao, Chunlin Luo, Qin Ma, Hao Wen, Jianhua Wang

**Affiliations:** ^1^Department of Pharmacognosy, Xinjiang Medical University, First Affiliated Hospital of Xinjiang Medical University, Urumqi 830011, Xinjiang, China; ^2^State Key Laboratory of Pathogenesis, Prevention, and Treatment of High Incidence Diseases in Central Asia, Xinjiang Medical University, First Affiliated Hospital of Xinjiang Medical University, Urumqi 830011, Xinjiang, China; ^3^Department of Pharmacy, Xinjiang Key Laboratory of Clinical Drug Research, First Affiliated Hospital of Xinjiang Medical University, Urumqi 830011, Xinjiang, China; ^4^Department of Pharmacognosy, School of Pharmacy, Xinjiang Medical University, Urumqi 830017, China; ^5^Department of Medicine, School of Pharmacy, Xinjiang Medical University, Urumqi 830017, China; ^6^Research and Development Department, Xinjiang Huashidan Drug Research Co. Ltd., Urumqi 830011, China

**Keywords:** Drp1, H-2-168, Mfn2, mitochondrial fusion and division, neurotoxicity, PC12 cells

## Abstract

**Background:** Harmine (HM) has several pharmacological effects; however, severe neurotoxicity limits its clinical application and development. HM neurotoxicity is associated with abnormal energy metabolism. This study aimed to explore the roles and underlying mechanisms of mitochondrial fusion and division in HM derivative H-2-168-induced neurotoxicity.

**Methods:** PC12 cells were treated with H-2-168, Mdivi-1 (an inhibitor of mitochondrial division), or a combination of both. Cell viability, levels of reactive oxygen species (ROS), adenosine triphosphate (ATP), lactic dehydrogenase (LDH), mitochondrial morphology, and membrane potential were measured. Immunofluorescence (IF) and western blotting were used to determine the expression of apoptosis-, mitochondrial fusion-, and division-related proteins. Additionally, PC12 cells with Drp1 knockdown or Mfn2 overexpression were generated to explore their effects.

**Results:** H-2-168 alone or in combination with Mdivi-1 significantly reduced PC12 cell viability, induced apoptosis, and impaired mitochondrial function. These effects were accompanied by increased levels of ROS and LDH, reduced ATP levels, upregulation of caspase-3, cytochrome c (Cyt-c), Drp1, and Fis1, and downregulation of Mfn2 and OPA1. Additionally, Drp1 knockdown or Mfn2 overexpression further enhanced the H-2-168-induced reduction in cell viability.

**Conclusions:** These data implied that H-2-168 may initiate apoptosis in PC12 cells by influencing the balance between mitochondrial fusion and division, accompanied by changes in energy metabolism, which may induce neurotoxicity.

## 1. Introduction

As the most important plant secondary metabolite group, β-carboline alkaloids are mainly distributed in *Peganum harmala* L. (the local herbal medicine of Xinjiang) and are widely used in the treatment of various diseases due to their significant pharmacological effects, such as anti-inflammatory, antibacterial, antitumor, and antihypertensive activities [1, 2]. Alkaloids are also responsible for the resistance against insect attack in plant–insect interactions [3]. These exogenous phytochemicals can act as attractants, repellents, or toxic substances to influence insect behavior and regulate their growth, development, and other physiological processes [4, 5].

Harmine (HM) is a natural β-carboline alkaloid that is abundant in the seeds of *P. harmala* L. As a therapeutic agent, HM has been proven to be the most effective compound in *P. harmala* L., owing to its antitumor and other pharmacological effects [6, 7]. The mechanisms through which HM exerts its therapeutic effects in human diseases are complex. HM exerts antitumor effects by inhibiting the activity of DNA topoisomerases I and II, intercalating into DNA, and interfering with DNA synthesis [8]. A previous study illustrated that HM induces DNA damage in *Echinococcus granulosus* by activating the EgATM-EgP53-EgTopo2a signaling pathway, thereby inducing *Echinococcus granulosus* death and exerting therapeutic effects for cystic echinococcosis (CE) [9]. Furthermore, HM is considered a potential candidate for the treatment of Alzheimer's disease as it inhibits acetylcholinesterase (AChE) and butyrylcholinesterase (BChE) activity [10]. Increasing evidence indicates that HM can directly interact with the catalytic active site of AChE, thus irreversibly inhibiting AChE [11, 12]. This indicates that HM induces severe neurotoxicity, including convulsions, tremors, neural inhibition, and excitation, which limits its clinical application and development [13, 14]. Because of its broad activity and simple tricyclic structure, HM has also been used as a lead compound for chemical structural transformations, and some HM derivatives with increased activity have been synthesized [15]. Our team, in cooperation with Xinjiang Huashidan Pharmaceutical (Xinjiang, China), designed and synthesized an HM derivative, H-2-168, which has the same parent nucleus as HM, and demonstrated that H-2-168 could be a very effective candidate compound for CE therapy with low toxicity [16]. However, the specific effects and underlying mechanisms by which the HM derivative H-2-168 induces neurotoxicity remain unclear.

HM neurotoxicity is reportedly related to abnormal energy metabolism [17]. The toxic effects of exogenous chemicals on neurocytes typically include mitochondrial toxicity, and the development of allergic symptoms is often closely associated with mitochondrial dysfunction [18]. Mitochondria are a constantly reorganizing dynamic network, and the balance between mitochondrial fusion and division is crucial for maintaining mitochondrial integrity and homeostasis, which play vital roles in regulating reactive oxygen species (ROS) generation, cellular metabolism, programmed cell death, energy production, and other basic cellular functions [19, 20]. Under normal physiological conditions, mitochondrial fusion and division are in dynamic equilibrium [21]. Disruption of this balance blocks mitochondrial fusion and division, leading to mitochondrial dysfunction and energy reduction, thereby inducing cell apoptosis [22]. An increasing number of studies suggest that mitochondrial dynamics, that is, restoring the balance between mitochondrial fusion and division, represent a powerful therapeutic target for neurotoxicity-related diseases [23, 24]. Dong et al. [25] demonstrated that 2,2′, 4,4′-tetrabromodiphenyl ether (PBDE-47) could disrupt mitochondrial dynamics, induce mitochondrial abnormalities, and trigger cell apoptosis, thus resulting in neuron loss and subsequent neurobehavioral deficits, which implied that targeting mitochondrial fusion division may be a promising therapeutic intervention against the neurotoxicity of PBDE-47. Another study showed that apigenin has a neuroprotective potential against lipopolysaccharide-induced neurotoxicity by regulating mitochondrial fusion and division-related proteins to maintain adequate mitochondrial homeostasis and function [26]. However, it is unclear whether the HM derivative H-2-168 induces neurocyte apoptosis by mediating mitochondrial fusion and division.

In this study, adrenal pheochromocytoma cells (PC12) were selected as nerve cells, and the HM derivative H-2-168 and mitochondrial division inhibitor (Mdivi-1) were used to treat PC12 cells. Cell viability and mitochondrial function were measured to explore the roles of the HM derivative H-2-168 and mitochondrial fusion and division in the growth of PC12 cells. Additionally, Mfn2 and Drp1 mainly participate in mitochondrial fusion and division and affect the speed of mitochondrial energy synthesis, thus affecting cell apoptosis. Therefore, we investigated the roles and potential mechanisms of Drp1 and Mfn2 in mitochondrial fusion and division in PC12 cells.

## 2. Materials and Methods

### 2.1. Cell Culture and Grouping

The nerve cell line PC12 was purchased from Wuhan Pricella Biotechnology Co., Ltd. (Wuhan, China) and maintained in Roswell Park Memorial Institute (RPMI) 1640 medium (HyClone, USA) containing 10% fetal bovine serum (FBS, Thermo Fisher Scientific, USA) and 1% penicillin/streptomycin (HyClone, USA). The PC12 cells were cultured in an incubator with 5% CO_2_ at 37°C, and the cells with the second or third passage were selected for subsequent experiments.

The PC12 cells were seeded into a 96-well plate and treated with different concentrations (0, 1, 10, 25, 50, and 100 μmol/L) of HM derivative H-2-168 (Xinjiang Huashidan Pharmaceutical Co. Ltd., Xinjiang, China), Mdivi-1 (MCE company, USA), and H-2-168: Mdivi-1 (1:1) for 24 h. Then, the cell viability was measured to choose the optimal concentrations of drugs. Additionally, the morphology of PC12 cells subjected to different treatments was observed under an optical microscope.

### 2.2. Cell Survival Assay

The viability of PC12 cells was tested using a 3-(4,5-dimethylthiazol-2-yl)-2,5-diphenyltetrazolium bromide (MTT) cell proliferation and cytotoxicity assay kit (Shanghai Yuanye Biotechnology Co., Ltd., Shanghai, China). Briefly, PC12 cells subjected to different treatments for 24 h were harvested, and the cell medium was discarded. The cells were washed with PBS three times, and then 10 μL MTT (5 mg/mL) reagent and 100 μL dimethylsulfoxide (DMSO) were added. After incubation for 4 h, the supernatant was removed, and 110 μL formazan solvent was added with low-speed oscillation for 10 min. After the purple crystals were completely dissolved, the absorbance (OD value) was measured at a wavelength of 490 nm using a microplate reader. The cell survival rate was calculated as follows: cell survival rate (%) = (OD_490 nm_ of drug treatment groups−OD_490 nm_ of blank control)/(OD_490 nm_ of the cell control group − OD_490 nm_ of blank control) × 100%.

### 2.3. Determination of ROS, Adenosine Triphosphate (ATP), and Lactic Dehydrogenase (LDH) in PC12 Cells

A ROS detection kit (Beyotime Biotechnology, Shanghai, China) was employed to determine the ROS levels in PC12 cells with 20 μmol/L HM derivative H-20168, Mdivi-1, and H-2-168: Mdivi-1 (1:1) in accordance with the manufacturer's instructions. In brief, the PC12 cells subjected to different treatments were washed with basal culture medium three times and then incubated with 0.5 mL 2′,7′-dichlorodihydrofluorescein diacetate (DCFH-DA) solution (DCFH-DA:medium = 1:1000). After incubation for 20 min, serum-free cell culture medium was used to wash the cells three times to remove the excess DCFH-DA, and the DCF fluorescence signal of the cells was observed and photographed using a confocal laser microscope (Leica, Germany).

An enhanced ATP assay kit (Beyotime Biotechnology) and an LDH assay kit (Solarbio, Beijing, China) were used to measure the levels of ATP and LDH in PC12 cells after different treatments according to the protocols of their corresponding manufacturers. For ATP measurement, PC12 cells with different treatments were washed three times with PBS, and the cell supernatant was collected. Then, 100 μL ATP lysis buffer was added, and the lytic cell solution was transferred to a new tube. After centrifugation at 12,000 rpm for 5 min at 4°C, the supernatant was added with 100 μL ATP detection working liquid. After incubation for 5 min, 20 μL samples or standard substance were added, and the relative light unit (RLU) values were immediately measured using a microplate reader. Furthermore, for the examination of LDH levels, the PC12 cells subjected to different treatments were washed with PBS, and then 150 μL LDH release reagent (PBS: LDH v:v = 10 : 1) was added. After mixing and incubating at 37°C for 1 h, the samples were centrifuged at 3000 rpm for 5 min, and 120 μL supernatant was used for the LDH examination.

### 2.4. Detection of Mitochondrial Morphology and Mitochondrial Membrane Potential in PC12 Cells

Mitochondrial morphology in PC12 cells with different treatments was observed using MitoTracker Red CMXRos (a red fluorescent probe; Beyotime Biotechnology). Briefly, PC12 cells were treated with different agents for 24 h and washed thrice with PBS. After removing the cell medium, the cells were incubated with MitoTracker Red CMXRos working solution (200 nM) at 37°C for 40 min. After removing the working solution, the cell medium at 37°C was added, as well as mitochondrial red fluorescence was observed under a laser scanning confocal microscope.

In addition, the mitochondrial membrane potential of PC12 cells subjected to different treatments was tested using a Mitochondrial Membrane Potential Assay Kit with JC-1 (Solarbio) following the manufacturer's recommendations. Briefly, PC12 cells subjected to different treatments were washed three times with PBS, and 1 mL of culture medium was added. Thereafter, 1 mL of JC-1 staining solution was added to the cells. After the samples were thoroughly mixed and incubated for 20 min, the supernatant was removed, and the cell pellets were washed three times with JC-1 dye buffer (1×). After sealing, images of PC12 cells were acquired under a fluorescence microscope.

### 2.5. Immunofluorescence (IF) Assay

The PC12 cells treated with 20 μmol/L HM derivative H-20168, Mdivi-1, and H-2-168: Mdivi-1 (1:1) were harvested and washed with PBS three times for 5 min each time, and then fixed with 4% paraformaldehyde for 15 min. After washing, the cells were incubated with 0.5% Triton X-100 at room temperature for 20 min for permeabilization and then incubated with 10% goat serum at 37°C for 30 min. Subsequently, the cells were incubated with the primary antibodies at 4°C overnight, and after 1 h of rewarming at room temperature, the cells were incubated with the fluorescent secondary antibody against rabbit or mouse at 37°C in the dark for 1 h. The primary antibodies used included anti-Drp1 (Abcam, Cambridge, UK), anti-caspase-3 antibody (Bioss, Beijing, China), and anti-Mfn2 antibody (Abcam, UK). After washing with PBS, the cells were stained with 4′,6-diamidino-2-phenylindole (DAPI) for 5 min, and images of the PC12 cells were acquired using an inverted fluorescent microscope. Finally, the fluorescence intensity was analyzed using Fiji software [27]. Briefly, three fields of view were randomly selected from the fluorescent sections of each group for image acquisition. The green channel of the images was segmented using the ImageJ software, and the fluorescent images were converted into 8-bit black-and-white images. The protein expression regions were selected according to the threshold, and the average grayscale value was calculated to represent the average fluorescence intensity.

### 2.6. Western Blotting

Total proteins were extracted from the PC12 cells administrated with 20 μmol/L HM derivative H-20168, Mdivi-1, and H-2-168: Mdivi-1 (1:1) using 100 μL lysis buffer (PMSF: RIPA = 1:100) with 5 min of ultrasound. After centrifugation at 12,000 rpm for 10 min, the cell supernatant was collected to determine protein concentration using a BCA assay kit. Subsequently, the protein samples (30 μg) were separated by 12.5% SDS–PAGE (upper: 80 V for 20 min; lower: 120 V for 1.5 h) and then transferred to PVDF membranes. After blocking with 5% skim milk for 1.5 h, the membranes were incubated with anti-OPA1 antibody (Bioss), anti-Bax antibody (Bioss), anti-Mfn2 antibody (Abcam), anti-cytochrome c (Cyt-c) antibody (Bioss), anti-Drp1 antibody (Abcam), anti-β-actin antibody (Bioss), and anti-Fis1 antibody (Bioss) at 4°C overnight. Next, the membranes were incubated with horseradish peroxidase (HPR)-labeled secondary antibodies (goat antimouse/rabbit IgG/HRP, 1:5000, Bioss) at room temperature for 1 h. Finally, the protein bands were developed using a Millipore electrochemical luminescence (ECL) assay kit (Thermo Fisher Scientific) and exposed to a chemiluminescence imager. Using β-actin as a reference protein, ImageJ software was used to calculate the gray value of each protein band.

### 2.7. Cell Transfection and Determination of Transfection Efficiency

To investigate the roles of Drp1 and Mfn2 in mitochondrial fusion and division in PC12 cells, PC12 cells with Drp1 knockdown or Mfn2 overexpression were constructed using small interfering RNAs (siRNA-1203, 1523, 2154) with different sequences (Table 1) or Mfn2 overexpressed (OE-Mfn2) plasmids through electroporation [28]. Briefly, the negative control (NC), siRNA-Drp1-1203, -1523, -2154, and OE-Mfn2 plasmids were synthesized and provided by GenePharma (Shanghai, China). The PC12 cells were cultured for 24 h, and 100 μL electroporation solution (200 mM glucose, 5 mM magnesium chloride, 2 mM hydrophobic ethanol, 20 mM Tris, pH = 7.4) contained about 10^5^ cells. Then, NC, siRNA-Drp1-1203, -1523, -2154, or OE-Mfn2 plasmids were added to the electroporation solution with the final concentration of 5 μM. After electric shock with a square wave in a 4 mm-electric shock cup at 125 V for 20 ms, the electric shock cup was immediately put into a 37°C constant temperature incubator containing 5% CO_2_ for 10 min and then transferred to a 96-well plate, including 1 mL complete medium for another 24 h of incubation. After transfection, the green fluorescence intensity in PC12 cells from each group was detected using an inverted fluorescence microscope, and the cell transfection efficiency was evaluated by determining Drp1 and Mfn2 expression in each group using real-time quantitative PCR (RT-qPCR) with Drp1 and Mfn2 sequences (Table 1). Additionally, the survival rate of the PC12 cells with different transfections was measured by MTT.

### 2.8. Statistical Analysis

Data are reported as mean ± standard deviation and statistical analysis was conducted in the SPSS software. Among them, Student's *t*-test was used to compare the differences between two groups, as well as for the comparison among more than two groups, and one-way analysis of variance followed by the Bonferroni method was applied. The statistical value of *p* less than 0.05 was regarded as a statistically significant difference. GraphPad Prism 5.0 (GraphPad Software, San Diego, CA, USA) was used for visualization.

## 3. Results

### 3.1. Screen of Optimal Concentrations of Drugs

**Figure 1:**
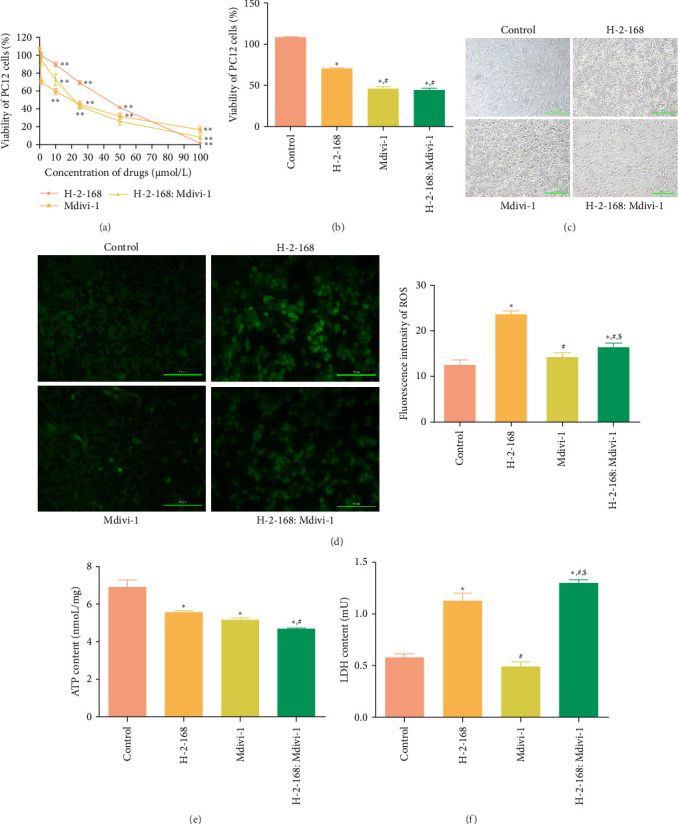
Screening of optimal drug concentrations and effects of H-2-168 on the growth of PC12 cells. (A) The viability of PC12 cells after being treated with different concentrations of drugs (0, 1, 10, 25, 50, and 100 μmol/L). *⁣*^*∗∗*^*p* < 0.01 vs. 0 μmol/L. (B) Viability of PC12 cells after treatment with different drugs. *⁣*^*∗*^*p* < 0.05 vs. control; ^#^*p* < 0.05 vs. H-2-168. (C) Morphology of PC12 cells treated with different drugs. (D) The levels of reactive oxygen species (ROS) in PC12 cells treated with the different drugs. (E) ATP levels in PC12 cells treated with different drugs. (F) Levels of lactic dehydrogenase (LDH) in PC12 cells treated with different drugs. *⁣*^*∗*^*p* < 0.05 vs. control; ^#^*p* < 0.05 vs. H-2-168; ^$^*p* < 0.05, vs. Mdivi-1.

To screen the optimal concentrations of drugs, different concentrations (0, 1, 10, 25, 50, and 100 μmol/L) of H-2-168, Mdivi-1, and H-2-168: Mdivi-1 (1:1) were used to treat PC12 cells, and then the cell viability was measured by MTT assay. It was found that with the increasing concentrations of the drugs, the viability of PC12 cells was gradually decreased significantly (*p* < 0.05), and the 24 h median effective concentrations (EC50) of H-2-168, Mdivi-1, and H-2-168: Mdivi-1 against PC12 cells were 37.02 μmol/L, 23.87 μmol/L, and 19.44 μmol/L, respectively (Figure 1A). Therefore, referring to the trend of cell viability, EC50 values, and drug action properties of each drug group, 20 μmol/L H-2-168, Mdivi-1, and H-2-168: Mdivi-1 were selected as the optimal concentrations to be administered to PC12 cells in subsequent experiments.

### 3.2. Effects of H-2-168 on Viability of PC12 Cells and Morphology Observation of PC12 Cells

PC12 cells were treated with the optimal concentrations of H-2-168, Mdivi-1, and H-2-168: Mdivi-1 and the MTT assay was used to determine cell viability. As shown in Figure 1B, compared with the control cells, the viability of PC12 cells was significantly inhibited by H-2-168 (*p* < 0.05), as well as was further evidently suppressed by Mdivi-1 and H-2-168: Mdivi-1 (*p* < 0.05). Subsequently, the morphology of cells subjected to different treatments was observed. In the control group, PC12 cells grew well in the resting state in the form of a long spindle or polygonal type. Some proliferating cells appeared as round or oval with transparency and strong refraction and extended to connect with other cells to form a network (Figure 1C). In the H-2-168, Mdivi-1, and H-2-168: Mdivi-1 groups, the adhesive properties of PC12 cells were decreased to varying degrees. Some cells began to detach, forming aggregates accompanied by decreased cell density and refraction and reduced or absent cell processes. Cell adhesion in the H-2-168 group declined slightly, and cell detachment was less. In contrast, cell adhesion in the Mdivi-1 group was significantly reduced, with most cells shrinking and detaching. Additionally, cells in the H-2-168: Mdivi-1 group showed less adhesion than those in the H-2-168 group, and some cells started to detach (Figure 1C). These results suggest that H-2-168 and the combination of H-2-168 and Mdivi-1 suppress the viability of PC12 cells.

### 3.3. Effects of H-2-168 on the Levels of ROS, ATP, and LDH in PC12 Cells

To further investigate the mechanism of action of H-2-168 in PC12 cell death, the levels of ROS, ATP, and LDH were determined. As shown in Figure 1D, DCF-labeled mitochondrial ROS showed green fluorescence, and the PC12 cells in the H-2-168 and H-2-168: Mdivi-1 groups displayed different degrees of green fluorescence. Green fluorescence in the Mdivi-1 group was not obvious. There was no significant difference in ROS levels between the control and Mdivi-1 groups (*p* > 0.05); however, compared to the control cells, ROS levels were evidently increased in the cells treated with H-2-168 and H-2-168: Mdivi-1 (*p* < 0.05, Figure 1D). The ATP content in the control, H-2-168, Mdivi-1, and H-2-168: Mdivi-1 groups was 6.86 ± 0.44 nmol/mg, 5.52 ± 0.13 nmol/mg, 5.12 ± 0.16 nmol/mg, and 4.64 ± 0.07 nmol/mg, respectively. This indicates that compared to the control cells, the ATP content in the PC12 cells administered with H-2-168, Mdivi-1, and H-2-168: Mdivi-1 were significantly reduced (*p* < 0.05), with the lowest ATP content observed in the H-2-168: Mdivi-1 group (Figure 1E). LDH level in the control, H-2-168, Mdivi-1, and H-2-168: Mdivi-1 groups was 0.57 ± 0.04, 1.12 ± 0.08, 0.48 ± 0.06, and 1.29 ± 0.04 mU, respectively. This indicates that no significant difference was found in the LDH level between the control and Mdivi-1 groups, while the LDH level was significantly increased in the H-2-168 group compared to the control cells (*p* < 0.05) and was significantly enhanced in the H-2-168: Mdivi-1 groups relative to the H-2-168-treated PC12 cells (*P* < 0.05, Figure 1F). The results suggested that H-2-168 and the combination of H-2-168 and Mdivi-1 increased the levels of ROS and LDH while decreasing the ATP level and that the combination of H-2-168 and Mdivi-1 had better effects on ATP and LDH levels.

### 3.4. Effects of H-2-168 on Mitochondrial Morphology and Mitochondrial Membrane Potential in PC12 Cells

**Figure 2:**
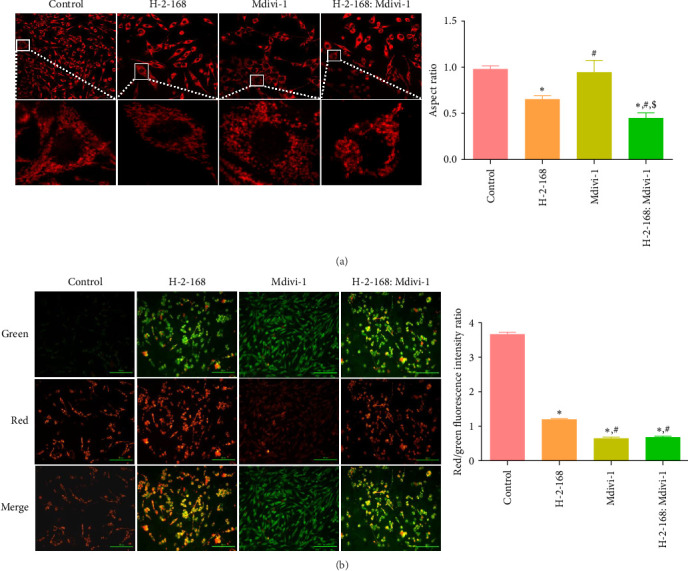
Effects of H-2-168 on mitochondrial morphology and mitochondrial membrane potential in PC12 cells. (A) Morphology and aspect ratio of mitochondria in PC12 cells treated with different drugs using MitoTracker Red CMXRos. (B) The mitochondrial membrane potential of PC12 cells after different treatments was tested using a Mitochondrial Membrane Potential Assay Kit with JC-1. *⁣*^*∗*^*p* < 0.05 vs. control; ^#^*p* < 0.05 vs. H-2–168; ^$^*p* < 0.05 vs. Mdivi-1.

We investigated the effects of H-2-168 on mitochondrial morphology and mitochondrial membrane potential in PC12 cells. As shown in Figure 2A, the mitochondria in control PC12 cells were normal, with long rod-like structures scattered in the cytoplasm. After treatment with H-2-168 or H-2-168: Mdivi-1, the mitochondria appeared fragmented, showing small points and short rods, and the number of fragmented mitochondria in PC12 cells treated with H-2-168: Mdivi-1 increased (Figure 2A). After calculating the aspect ratio of mitochondria in the different groups, we observed that compared to the control cells, Mdivi-1 did not significantly change the aspect ratio of mitochondria (*p* > 0.05), whereas H-2-168 and H-2-168: Mdivi-1 evidently reduced the aspect ratio of mitochondria (*p* < 0.05, Figure 2A). Furthermore, the aspect ratio of the mitochondria in the H-2-168: Mdivi-1 group tended to be 1, suggesting that the degree of mitochondrial fragmentation was severe, and the shape tended to be circular. Subsequently, the mitochondrial membrane potential of the PC12 cells with different treatments was examined using the JC-1 assay. Compared to the control PC12 cells, the red/green fluorescence intensity ratio was significantly reduced in the H-2-168, Mdivi-1, and H-2-168: Mdivi-1 groups (*p* < 0.05), and further decreased in the H-2-168: Mdivi-1 group compared to the H-2-168-induced cells (*p* < 0.05, Figure 2B). These results suggest that H-2-168 and the combination of H-2-168 and Mdivi-1 could damage the mitochondrial function of PC12 cells, with the combination showing greater efficacy.

### 3.5. Effects of H-2-168 on Apoptosis-Related Proteins and Mitochondrial Fusion Division-Related Proteins in PC12 Cells

**Figure 3:**
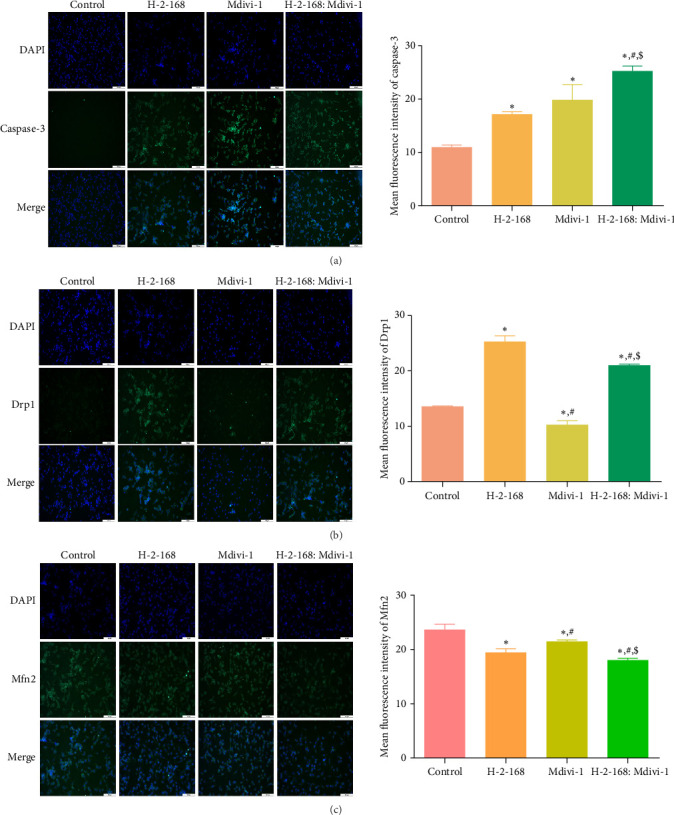
Expression of apoptosis-related and mitochondrial fusion division-related proteins in PC12 cells subjected to different treatments using immunofluorescence. (A) The mean fluorescence intensity of caspase-3 in PC12 cells treated with different drugs. (B) The mean fluorescence intensity of Drp1 in PC12 cells treated with different drugs. (C) The mean fluorescence intensity of Mfn2 in PC12 cells treated with different drugs. *⁣*^*∗*^*p* < 0.05 vs. control; ^#^*p* < 0.05 vs. H-2-168; ^$^*p* < 0.05 vs. Mdivi-1.

To investigate the molecular mechanism by which H-2-168 induces PC12 cell death, the expression of apoptosis-related proteins (caspase-3 and Cyt-c) and mitochondrial fusion division-related proteins (Drp1, Mfn2, Fis1, and OPA1) was determined using IF and western blotting. The IF results showed that the caspase-3 levels in the PC12 cells treated with H-2-168, Mdivi-1, and H-2-168: Mdivi-1 were significantly higher than those in the control cells (*p* < 0.05), with the highest level in the H-2-168: Mdivi-1-administrated PC12 cells (Figure 3A). Relative to control cells, the fluorescence intensity of Drp1 was significantly increased in H-2-168-treated or H-2-168: Mdivi-1-treated cells (*p* < 0.05), whereas it was evidently decreased in Mdivi-1-induced PC12 cells (*p* < 0.05, Figure 3B). For Mfn2, its fluorescence intensity was significantly lower in the H-2-168 group than that in the control group (*p* < 0.05), whereas it was significantly lower in the H-2-168: Mdivi-1 group (*p* < 0.05, Figure 3C).

**Figure 4:**
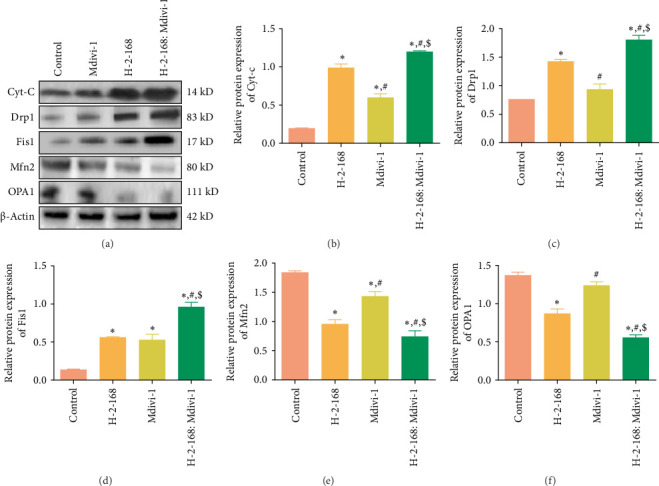
Expression of apoptosis-related and mitochondrial fusion division-related proteins in PC12 cells after different treatments using western blotting. (A) Representative western blots. Protein expression of Cyt-c (B), Drp1 (C), Fis1 (D), Mfn2 (E), and OPA1 (F) in PC12 cells subjected to different treatments. *⁣*^*∗*^*p* < 0.05 vs. control; ^#^*p* < 0.05 vs. H-2-168; ^$^*p* < 0.05 vs. Mdivi-1.

Furthermore, western blotting results showed that treatment with H-2-168, Mdivi-1, and H-2-168: Mdivi-1 significantly upregulated Cyt-c expression relative to that in control PC12 cells (*p* < 0.05), and the combination of H-2-168 and Mdivi-1 had the best action (Figure 4A,B). The mitochondrial division-related proteins (Drp1 and Fis1) were significantly upregulated in the H-2-168 and H-2-168: Mdivi-1-administered PC12 cells compared to those in the control PC12 cells (*p* < 0.05), and were highest in the H-2-168: Mdivi-1-administered PC12 cells (Figure 4A,C,D). For the mitochondrial fusion-related proteins Mfn2 and OPA1, expression levels were significantly reduced in all drug-treated groups compared with control cells (*p* < 0.05). Furthermore, expression was even lower in PC12 cells treated with the combination of H-2-168 and Mdivi-1 compared to cells treated with either drug alone (*p* < 0.05; Figure 4A,E,F).

### 3.6. Selection of Drp1 Interference Sequences and Transfection Efficiency

**Figure 5:**
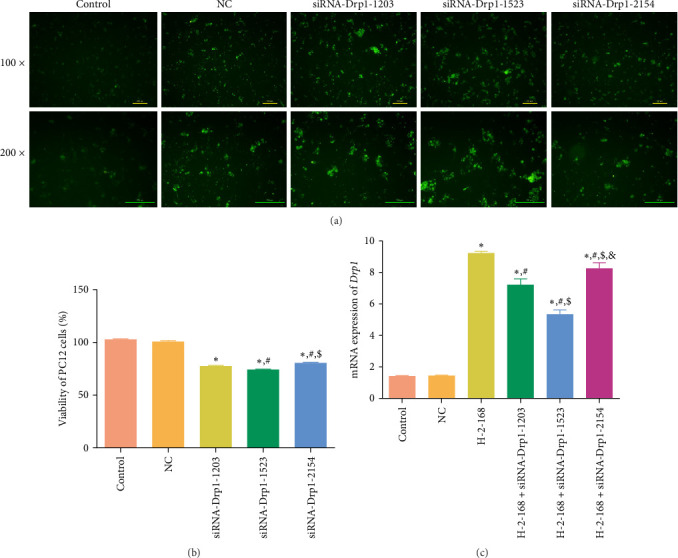
Selection of interference sequences and assessment of transfection efficiency of Drp1. (A) Green fluorescence in PC12 cells after electroporation with different small interference RNAs (siRNAs) was observed using an inverted fluorescence microscope. (B) Viability of PC12 cells transfected with different siRNAs. *⁣*^*∗*^*p* < 0.05 vs. control; ^#^*p* < 0.05 vs. siRNA-Drp1-1203; ^$^*p* < 0.05 vs. siRNA-Drp1-1523. (C) The mRNA expression of *Drp1* in PC12 cells after transfection with different siRNAs was examined by RT-qPCR. *⁣*^*∗*^*p* < 0.05 vs. control; ^#^*p* < 0.05 vs. H-2-168; ^$^*p* < 0.05 vs. H-2-168 + siRNA-Drp1-1203; ^&^*p* < 0.05 vs. H-2-168 + siRNA-Drp1-1523.

Drp1 is a mitochondrial division-related protein and is reported to be associated with cellular mitochondrial fusion and division. Our aforementioned results showed that Drp1 was significantly upregulated in H-2-168-induced and H-2-168: Mdivi-1-treated PC12 cells. Therefore, we further constructed PC12 cells with Drp1 knockdown by electroporation to explore the role of Drp1 in the growth of PC12 cells. After transfection, the PC12 cells in the control group showed no green fluorescent spots, whereas the PC12 cells in the NC, siRNA-Drp1-1203, -1523, and-2154 groups showed varying degrees of bright green fluorescent spots; the green fluorescent spots were stronger in the siRNA-Drp1-1523 group than in the other groups (Figure 5A), which implied that these interference sequences with green fluorescence could be successfully introduced into PC12 cells by electroporation. The cell viability was determined after transfection. No significant difference in the viability of PC12 cells was found between the control and NC groups (*p* > 0.05). However, after transfection with siRNA-Drp1-1203, -1523, and -2154, the viability of PC12 cells was significantly inhibited compared to that of the control cells (*p* < 0.05), with better effects of siRNA-Drp1-1523 (Figure 5B). RT-qPCR was performed to measure Drp1 expression following transfection. The expression of *Drp1* in the control and NC groups were respectively 1.36 ± 0.08 and 1.39 ± 0.10, which showed no significant difference (*p* > 0.05, Figure 5C). After H-2-168 treatment, the *Drp1* expression (9.15 ± 0.18) was significantly higher than that in the control group (*p* < 0.05), but compared to the H-2-168 group, the *Drp1* expression after H-2-168 + siRNA-Drp1-1203, -1523, and -2154 transfection were upregulated, and the *Drp1* expression in the H-2-168 + siRNA-Drp1-1523 was significantly lower than that in the H-2-168 + siRNA-Drp1-1203, and -2154 groups (*p* < 0.05, Figure 5C). Therefore, in further experiments, siRNA-Drp1-1523 was selected, and the PC12 cells with Drp1 knockdown were successfully established.

### 3.7. Effects of Drp1 on PC12 Cell Viability and on the Expression of Related Proteins

**Figure 6:**
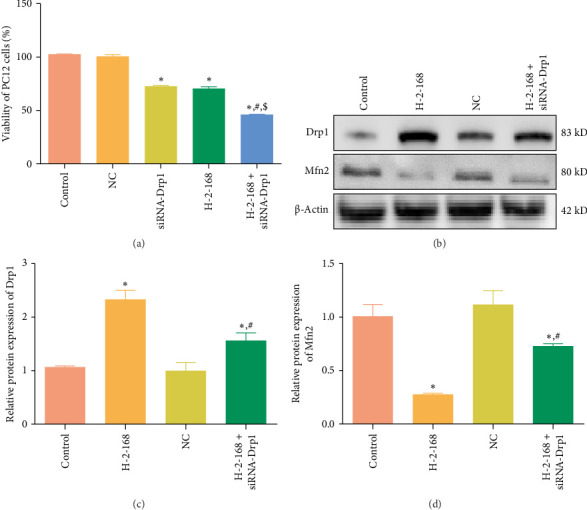
Effects of Drp1 on the viability of PC12 cells and expression of related proteins. (A) The viability of PC12 cells with H-2-168 treatment and siRNA-Drp1 transfection using MTT. *⁣*^*∗*^*p* < 0.05 vs. control; ^#^*p* < 0.05 vs. siRNA-Drp1; ^$^*p* < 0.05 vs. H-2-168. (B) Representative western blotting bands of PC12 cells transfected with H-2-168 treatment and siRNA-Drp1 transfection. (C) The protein expression of Drp1 in PC12 cells treated with H-2-168 treatment and siRNA-Drp1 transfection. (D) The protein expression of Mfn2 in PC12 cells treated with H-2-168 treatment and siRNA-Drp1 transfection. *⁣*^*∗*^*p* < 0.05 vs. control; ^#^*p* < 0.05 vs. H-2-168.

The viability of PC12 cells in the control, NC, siRNA-Drp1, H-2-168, and H-2-168 + siRNA-Drp1 were 101.99% ± 0.32%, 99.73% ± 2.35%, 71.66% ± 0.87%, 69.37% ± 2.55%, and 44.76% ± 0.78%, respectively. These results showed that compared to the control group, the viability of PC12 cells was significantly reduced by siRNA-Drp1 and H-2-168 (*p* < 0.05), and the combination of H-2-168 and siRNA-Drp1 further reduced the viability of PC12 cells (*p* < 0.05, Figure 6A). These data suggested that the *Drp1* knockout reduced the tolerance of PC12 cells to the HM derivative H-2-168.

Western blotting was performed to detect Drp1 and Mfn2 protein levels after transfection. There were no significant differences in Drp1 or Mfn2 protein expression between the control and NC groups (*p* > 0.05; Figure 6B–D). Relative to the control cells, H-2-168 significantly upregulated Drp1 expression (*P* < 0.05) and markedly downregulated Mfn2 expression (*p* < 0.05, Figure 6B–D). However, siRNA-Drp1 interference partially restored the levels of Drp1 and Mfn2 induced by H-2-168 (*p* < 0.05; Figure 6B–D).

### 3.8. Effects of Mfn2 on Viability of PC12 Cells and on the Expression of Mfn2 and Drp1

**Figure 7:**
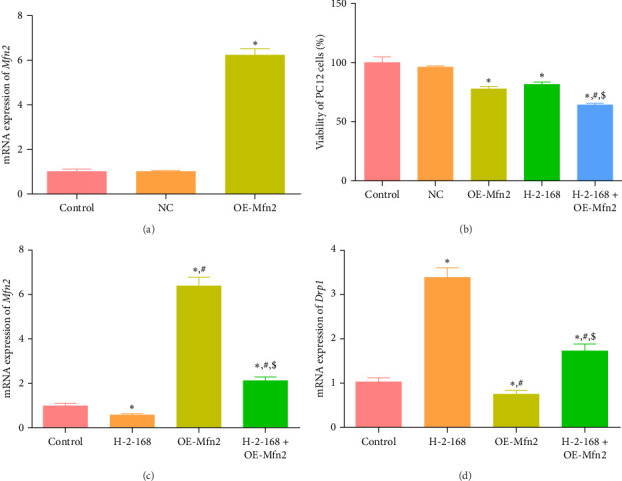
Effects of Mfn2 on the viability of PC12 cells and expression of *Drp1* and *Mfn2*. (A) Cell transfection efficiency was assessed by measuring Mfn2 mRNA expression using RT-qPCR. *⁣*^*∗*^*p* < 0.05 vs. control. (B) The viability of PC12 cells transfected with OE-Mfn2 and treated with H-2-168. *⁣*^*∗*^*p* < 0.05 vs. control; ^#^*p* < 0.05 vs. OE-Mfn2; ^$^*p* < 0.05 vs. H-2-168. The mRNA expression of *Mfn2* (C) and *Drp1* (D) in PC12 cells after transfection with OE-Mfn2 and H-2-168 was examined by RT-qPCR. *⁣*^*∗*^*p* < 0.05 vs. control; ^#^*p* < 0.05 vs. H-2-168; ^$^*p* < 0.05 vs. OE-Mfn2.

Mfn2 is a mitochondrial fusion-related protein closely related to cellular mitochondrial fusion and division. In addition, our results showed a lower expression of Mfn2 after treatment with H-2-168 and H-2-168: Mdivi-1s. Therefore, PC12 cells overexpressing Mfn2 were established by electroporation to investigate the effects of Mfn2 on PC12 growth. OE-Mfn2 transfection significantly elevated *Mfn2* expression compared to that in the control and NC-transfected PC12 cells (*p* < 0.05, Figure 7A), indicating that PC12 cells overexpressing Mfn2 were successfully constructed and could be used for further experiments.

After that, it was obvious that there was no significant difference in the viability of PC12 cells between the control and NC groups (*p* > 0.05). Compared to the control cells, OE-Mfn2 and H-2-168 alone significantly inhibited the viability of PC12 cells (*P* < 0.05), and OE-Mfn2 combined with H-2-168 further reduced the viability of PC12 cells compared to the OE-Mfn2 or H-2-168 groups (*p* < 0.05, Figure 7B). Additionally, the mRNA expression levels of *Mfn2* and *Drp1* were measured. Compared with the control PC12 cells, H-2-168 downregulated *Mfn2* expression while upregulating *Drp1* expression (*p* < 0.05); however, Mfn2 overexpression markedly upregulated *Mfn2* expression while downregulating *Drp1* expression (*p* < 0.05, Figure 7C,D). Compared to the H-2-168 group, Mfn2 expression was significantly higher, whereas Drp1 expression was lower in the H-2-168 + OE-Mfn2 group (*p* < 0.05; Figure 7C,D).

## 4. Discussion

HM is extracted from a local herb in Xinjiang and has good pharmacological activity [29]. It has been established that HM and its derivatives have lower hepatotoxicity and higher anti-insect activity than albendazole for clinical use [30], but their clinical application is limited owing to their toxic effects on the central nervous system. Therefore, exploring the neurotoxic mechanisms of HM and their derivatives is important. Liu et al. [31] demonstrated that HM and its derivatives induce cell apoptosis by activating the cellular mitochondrial pathway. Moreover, we recently observed that HM neurotoxicity is related to abnormal energy metabolism [32]. Our research group previously found that mitochondrial dynamics contribute to HM-induced damage in PC12 cells and initiate mitochondrial apoptosis [33]. However, whether the neurotoxicity of the HM derivative, H-2-168, is mediated by mitochondrial fusion division remains unknown. Our research showed that the combination of H-2-168 and Mdivi-1 more effectively reduced PC12 cell viability, induced apoptosis, and impaired mitochondrial function. These effects were accompanied by increased ROS and LDH levels, reduced ATP content, upregulation of caspase-3, Cyt-c, Drp1, and Fis1, and downregulation of Mfn2 and OPA1. Furthermore, Drp1 knockdown or Mfn2 overexpression exacerbated the H-2-168-induced decrease in PC12 cell viability. These data imply that H-2-168 may initiate apoptosis in PC12 cells by influencing the balance between mitochondrial fusion and division, accompanied by changes in energy metabolism, which may induce neurotoxicity.

PC12 cells are commonly used to study the neurotoxic activity of various substances from natural or synthetic processes by evaluating their effects on cell viability, apoptosis, DNA damage, neurite growth, and protein expression levels [34]. Huang et al. [35] showed that Aflatoxin B1 inhibits cell proliferation while promoting apoptosis by inducing ROS accumulation, S-phase cell cycle arrest, and DNA damage, thereby exhibiting extensive cytotoxicity in neuronal cells. Another study used PC12 cells to observe that compared with each drug alone, the combination of methamphetamine and heroin significantly reduced cell viability, accelerated cell apoptosis, and significantly reduced mitochondrial potential, indicating that the combination of methamphetamine and heroin may be more neurotoxic [36]. Additionally, Mdivi-1 is a mitochondrial division inhibitor that alleviates cardiac fibrosis in the infarct boundary area after infarction by inhibiting Drp1-activated mitochondrial division and oxidative stress [37]. The current study showed that the combination of H-2-168 and Mdivi-1 could significantly inhibit the growth of PC12 cells and accelerate their apoptosis, suggesting that the HM derivative H-2-168 may play neurotoxic roles by affecting the mitochondrial fusion and division pathways.

Mitochondria are the power sources of cells and participate in basic cellular functions, including ATP production, ROS signaling, intracellular calcium regulation, cell survival, and apoptosis [38]. The balance between mitochondrial fusion and division supports the metabolic function of mitochondria, thus providing power for various physiological processes in cells, such as cell growth, migration, and maintenance of redox homeostasis [39]. Additionally, the dynamic balance between mitochondrial fusion and division not only maintains mitochondrial function but also serves as a checkpoint for cell growth [40]. However, abnormal mitochondrial fusion and division contribute to mitochondrial dysfunction and changes in energy metabolism [41]. Our results show that the combination of H-2-168 and Mdivi-1 increased ROS and LDH levels, decreased ATP levels, altered mitochondrial morphology, and reduced mitochondrial levels. ROS are byproducts of cellular aerobic respiration and play crucial roles in cell death, including apoptosis, autophagy, and ferroptosis [42]. ROS levels increase after oxidative damage or stress in the mitochondria [43]. ATP is a central metabolite produced by the mitochondria through oxidative phosphorylation and plays an indispensable role in various cellular processes, from energy supply to intercellular signaling [44]. A previous study showed that adiponectin can suppress mitochondrial fusion and division by increasing ATP content and mitochondrial membrane potential and reducing ROS content, thereby improving chronic intermittent hypoxia-induced pancreatic injury [45]. In cells, pyruvate produced by glycolysis is converted to lactic acid by lactate LDH under anaerobic conditions, a process that can disrupt mitochondrial homeostasis [46]. The integrity of the mitochondrial morphology and normal mitochondrial membrane potential are commonly used to reflect mitochondrial homeostasis. An imbalance in mitochondrial homeostasis may result in the overproduction of ROS, and excessive ROS levels can induce the opening of mitochondrial transition pores, leading to impaired mitochondrial membrane potential and ultimately aggravating injury [47]. Gao et al. [48] showed that hypoxic mesenchymal stem cell-derived exosomes could ameliorate mitochondrial fatty acid oxidation to relieve ischemia-reperfusion injury by improving mitochondrial homeostasis, which is characterized by repairing the mitochondrial structure, inhibiting ROS, restoring mitochondrial ATP production, and increasing mitochondrial membrane potential. Another study demonstrated that Mdivi-1 could enhance ovarian function by mitigating mitochondrial dysfunction (increased ATP and mitochondrial membrane potential, as well as reduced ROS levels) in ovarian granulosa cells [49]. Taken together, we speculate that the combination of H-2-168 and Mdivi-1 may repress the growth of PC12 cells by altering the morphology of mitochondria and damaging mitochondrial function (increased ROS and LDH levels and decreased ATP content and mitochondrial membrane potential).

In addition, we explored the expression of apoptosis-related and mitochondrial fusion and division-related proteins and found that the combination of H-2-168 and Mdivi-1 further upregulated caspase-3, Cyt-c, Drp1, and Fis, whereas it downregulated Mfn2 and OPA1 expression. Caspase-3, an important protein associated with apoptosis, plays essential roles in normal neuronal development and neuropathology [50]. Hosseini et al. [51] demonstrated that linalool alleviated oxygen and glucose deprivation/reoxygenation (OGD/R)-induced injury in PC12 cells by inhibiting caspase-3 and caspase-9 during apoptosis. Cyt-c is an electron carrier in the mitochondrial respiratory chain. Its dysregulation can lead to abnormal functioning of the respiratory chain, causing ATP deficiency and initiation of apoptosis [52]. Drp1 and Fis are the only two proteins that are evolutionarily conserved during mitochondrial division and that interact with each other to promote mitochondrial division [53]. A previous research of Song et al. [54] showed that Drp1-mediated dysfunction of mitochondrial dynamics could contribute to renal injury, and suppression of the Drp1-Fis1 interaction could reduce abnormal mitochondrial fragmentation and acute kidney injury. Additionally, it has been reported that mitochondrial fusion is overactivated in tumor organoids, as well as in cancer; enhancing mitochondrial fusion alters metabolism and promotes tumor cell growth, whereas blocking mitochondrial fusion reduces oxygen consumption and ATP production in tumor cells [55]. Mfn2 and OPA1 are two common mitochondrial fusion-associated proteins, and their upregulation can reduce oxidative stress and mitochondrial damage, restore mitochondrial function, and delay lung cell senescence [56]. These reports, combined with our results, suggest that H-2-168 induces apoptosis of PC12 cells by regulating apoptosis-related proteins (caspase-3 and Cyt-c) and mitochondrial fusion division-related proteins (Drp1, Fis1, OPA1, and Mfn2), thereby exhibiting neurotoxicity.

An increasing number of studies have emphasized that mitochondrial dynamics are closely related to metabolic health and cell homeostasis [19, 57]. Drp1 and Mfn2 are fundamental core regulators of mitochondrial fusion and division. We established PC12 cells with Drp1 knockdown or Mfn2 overexpression and found that the viability of PC12 cells was further reduced after Drp1 knockdown or Mfn2 overexpression compared to that of H-2-168 cells. Chang et al. [58] reported that AgNPs promote mitochondria-dependent apoptosis of HT22 cells through the ROS-Drp1-mitochondrial division axis, thereby inducing mitochondrial damage and cytotoxicity in HT22 cells. Another study indicated that Drp1 deficiency could inhibit the activation and proliferation of TGF-β1-induced renal interstitial fibroblasts, whereas facilitate cell apoptosis, as well as reduce mitochondrial breakdown, ROS elevation, and glycolytic metastasis after TGF-β1 stimulation, which implied that Drp1 may be a therapeutic target for delaying the development of chronic kidney disease [59]. Therefore, we hypothesize that Drp1-mediated mitochondrial division and Mfn2-mediated mitochondrial fusion may be involved in H-2-168-induced neurotoxicity in PC12 cells.

However, this study had some limitations. First, the specific roles and mechanisms of Drp1 and Mfn2 in H-2-168-induced neurotoxicity in PC12 cells require further verification. Additionally, whether the detrimental effects of H-2-168 are directly mediated through mitochondrial dysfunction and the relationships among H-2-168, mitochondrial dynamics, and cell viability should be further investigated.

In conclusion, the HM derivative, H-2-168, induced mitochondrial injury and cytotoxicity in PC12 cells. H-2-168 may contribute to the apoptosis of PC12 cells by promoting mitochondrial fragmentation and dysfunction (increased ROS and LDH levels while decreasing ATP content and mitochondrial membrane potential), as well as by regulating apoptosis-related (caspase-3 and Cyt-c) and mitochondrial fusion division-related proteins (Drp1, Fis1, OPA1, and Mfn2). Additionally, Drp1 and Mfn2 have prominent effects on mitochondrial injury, and Drp1/Mfn2-mediated mitochondrial fusion and division may inhibit the growth of PC12 cells and promoting the neurotoxicity of H-2-168. This study contributes to our understanding of the roles of mitochondrial fusion and division in H-2-168-induced neurotoxicity and lays a theoretical foundation for Drp1/Mfn2-mediated mitochondrial fusion and division as possible alternative targets for the prevention and treatment of H-2-168-induced neurotoxicity.

## Figures and Tables

**Table 1:** The sequences of all primers.

Primer	Sequences (5′-3′)
Drp1-siRNA-1203	Sense: CUCCAGCUUAUUACCAAAUTT Antisense: AUUUGGUAAUAAGCUGGAGTT

Drp1-siRNA-1523	Sense: GAGGAUCAUUCAGCAUUGUTT Antisense: ACAAUGCUGAAUGAUCCUCTT

Drp1-siRNA-2154	Sense: GGGCAGCUGUAUAAGUCAUTT Antisense: AUGACUUAUACAGCUGCCCTT

Negative control	Sense: UUCUUCGAACGUGUCACGUTT Antisense: ACGUGACACGUUCGGAGAATT

Drp1	F: GTTCCTACAGTCCATCCTAAT R: CTCCATCAAGCCAGCATT

Mfn2	F: CTGCTAGGAGTTGCTGCATATAA
	R: TCAGCCATGTGTCGCTTATC

β-actin	F: CTACCTCATGAAGATCCTGACC R: CACAGCTTCTCTTTGATGTCAC

## Data Availability

The dataset used and/or analyzed during the current study is available from the corresponding author upon a reasonable request.

## References

[1] Hamsa T. P., Kuttan G. (2010). Harmine Inhibits Tumour Specific Neo-Vessel Formation by Regulating VEGF, MMP, TIMP and Pro-Inflammatory Mediators Both in Vivo and in Vitro. *European Journal of Pharmacology*.

[2] Uhl K. L., Schultz C. R., Geerts D., Bachmann A. S. (2018). Harmine, a Dual-Specificity Tyrosine Phosphorylation-Regulated Kinase (DYRK) Inhibitor Induces Caspase-Mediated Apoptosis in Neuroblastoma. *Cancer Cell International*.

[3] Bubica Bustos L. M., Ueno A. C., Di Leo T. D. (2020). Maternal Exposure to Ozone Modulates the Endophyte-Conferred Resistance to Aphids in *Lolium multiflorum* Plants. *Insects*.

[4] Palmer-Young E. C., Sadd B. M., Stevenson P. C., Irwin R. E., Adler L. S. (2016). Bumble Bee Parasite Strains Vary in Resistance to Phytochemicals. *Scientific Reports*.

[5] Popay A. J., Jensen J. G., Simpson W. R., Mace W. J., Somchit C. (2023). Translocation of Loline Alkaloids in Epichloë-Infected Cereal and Pasture Grasses: What the Insects Tell Us. *Journal of Fungi*.

[6] Zhang L., Li D., Yu S. (2020). Pharmacological Effects of Harmine and its Derivatives: A Review. *Archives of Pharmacal Research*.

[7] Zheng Z. H., Lin X. C., Lu Y. (2023). Harmine Exerts Anxiolytic Effects by Regulating Neuroinflammation and Neuronal Plasticity in the Basolateral Amygdala. *International Immunopharmacology*.

[8] Geng X., Ren Y., Wang F. (2018). Harmines Inhibit Cancer Cell Growth Through Coordinated Activation of Apoptosis and Inhibition of Autophagy. *Biochemical and Biophysical Research Communications*.

[9] Lu S., Wen L., Gong Y. (2021). In Vitro Effects of Harmine Against Echinococcus Granulosus Protoscoleces by Stimulating DNA Damage. *Experimental Parasitology*.

[10] Li S. P., Wang Y. W., Qi S. L. (2018). Analogous *β*-Carboline Alkaloids Harmaline and Harmine Ameliorate Scopolamine-Induced Cognition Dysfunction by Attenuating Acetylcholinesterase Activity, Oxidative Stress, and Inflammation in Mice. *Frontiers in Pharmacology*.

[11] Du H., Song J., Ma F. (2023). Novel Harmine Derivatives as Potent Acetylcholinesterase and Amyloid Beta Aggregation Dual Inhibitors for Management of Alzheimer’s Disease. *Journal of Enzyme Inhibition and Medicinal Chemistry*.

[12] Lv Y., Liang H., Li J. (2021). Central Inhibition Prevents the in Vivo Acute Toxicity of Harmine in Mice. *The Journal of Toxicological Sciences*.

[13] Hu Y., Yu X., Yang L. (2024). Research Progress on the Antitumor Effects of Harmine. *Frontiers in Oncology*.

[14] Li S., Wang A., Gu F. (2015). Novel Harmine Derivatives for Tumor Targeted Therapy. *Oncotarget*.

[15] Zhong G., Cui G., Yi X., Sun R., Zhang J. (2016). Insecticide Cytotoxicology in China: Current Status and Challenges. *Pesticide Biochemistry and Physiology*.

[16] Chen B., Yan M., Gao H. (2023). In Vitro and in Vivo Efficacies of Novel Harmine Derivatives in the Treatment of Cystic Echinococcosis. *Drug Design, Development and Therapy*.

[17] Zhang J., Zhang Z., Shu B., Cui G., Zhong G. (2018). Cytotoxic and Apoptotic Activity of the Novel Harmine Derivative ZC-14 in Sf9 Cells. *International Journal of Molecular Sciences*.

[18] Yu J., Liufu T., Zheng Y. (2022). CGG Repeat Expansion in NOTCH2NLC Causes Mitochondrial Dysfunction and Progressive Neurodegeneration in Drosophila Model. *Proceedings of the National Academy of Sciences*.

[19] Adebayo M., Singh S., Singh A. P., Dasgupta S. (2021). Mitochondrial Fusion and Fission: The Fine-Tune Balance for Cellular Homeostasis. *The FASEB Journal*.

[20] Annesley S. J., Fisher P. R. (2019). Mitochondria in Health and Disease. *Cells*.

[21] Giacomello M., Pyakurel A., Glytsou C., Scorrano L. (2020). The Cell Biology of Mitochondrial Membrane Dynamics. *Nature Reviews Molecular Cell Biology*.

[22] Chan D. C. (2020). Mitochondrial Dynamics and Its Involvement in Disease. *Annual Review of Pathology: Mechanisms of Disease*.

[23] Gao J., Luo A., Yan J. (2018). Mdivi-1 Pretreatment Mitigates Isoflurane-Induced Cognitive Deficits in Developmental Rats. *American Journal of Translational Research*.

[24] Shah A. J., Mir P. A., Adnan M. (2023). Synthetic and Natural Bioactive Molecules in Balancing the Crosstalk Among Common Signaling Pathways in Alzheimer’s Disease: Understanding the Neurotoxic Mechanisms for Therapeutic Intervention. *ACS Omega*.

[25] Dong L., Li P., Yang K. (2020). Promotion of Mitochondrial Fusion Protects Against Developmental PBDE-47 Neurotoxicity by Restoring Mitochondrial Homeostasis and Suppressing Excessive Apoptosis. *Theranostics*.

[26] Ahmedy O. A., Abdelghany T. M., El-Shamarka M. E. A., Khattab M. A., El-Tanbouly D. M. (2022). Apigenin Attenuates LPS-Induced Neurotoxicity and Cognitive Impairment in Mice via Promoting Mitochondrial Fusion/Mitophagy:Role of SIRT3/PINK1/Parkin Pathway. *Psychopharmacology*.

[27] Hulsey-Vincent H., Alvinez N., Witus S., Kowalski J. R., Dahlberg C. (2023). A Fiji Process for Quantifying Fluorescent Puncta in Linear Cellular Structures. *MicroPublication Biology*.

[28] Liu J., Jiang J., He M. (2023). Nanopore Electroporation Device for DNA Transfection Into Various Spreading and Nonadherent Cell Types. *ACS Applied Materials & Interfaces*.

[29] Khan H., Patel S., Kamal M. A. (2017). Pharmacological and Toxicological Profile of Harmane-*β*-Carboline Alkaloid: Friend or Foe. *Current Drug Metabolism*.

[30] Gong Y., Lv S., Tian C. (2020). Effect of Harmine and its Derivatives Against *Echinococcus granulosus* and Comparison of DNA Damage Targets. *Journal of Biomedical Nanotechnology*.

[31] Liu J., Li Q., Liu Z. (2016). Harmine Induces Cell Cycle Arrest and Mitochondrial Pathway-Mediated Cellular Apoptosis in SW620 Cells via Inhibition of the Akt and ERK Signaling Pathways. *Oncology Reports*.

[32] Liu Z., Lv J., Zhang Z. (2021). The Main Mechanisms of Trimethyltin Chloride-Induced Neurotoxicity: Energy Metabolism Disorder and Peroxidation Damage. *Toxicology Letters*.

[33] Gong Y., Zhao M., Ma R., Lin Y., Zhao J., Wang J. (2024). Effect of Harmine on Mitochondrial Fusion and Division During Apoptosis of PC12 Cells. *Herald of Medicine*.

[34] Wiatrak B., Kubis-Kubiak A., Piwowar A., Barg E. (2020). PC12 Cell Line: Cell Types, Coating of Culture Vessels, Differentiation and Other Culture Conditions. *Cells*.

[35] Huang B., Chen Q., Wang L. (2020). Aflatoxin B1 Induces Neurotoxicity Through Reactive Oxygen Species Generation, DNA Damage, Apoptosis, and S-Phase Cell Cycle Arrest. *International Journal of Molecular Sciences*.

[36] Tian X., Ru Q., Xiong Q. (2017). Neurotoxicity Induced by Methamphetamine-Heroin Combination in PC12 Cells. *Neuroscience Letters*.

[37] Ding J., Zhang Z., Li S. (2022). Mdivi-1 Alleviates Cardiac Fibrosis Post Myocardial Infarction at Infarcted Border Zone, Possibly via Inhibition of Drp1-Activated Mitochondrial Fission and Oxidative Stress. *Archives of Biochemistry and Biophysics*.

[38] Bhatti J. S., Bhatti G. K., Reddy P. H. (2017). Mitochondrial Dysfunction and Oxidative Stress in Metabolic Disorders–A Step Towards Mitochondria Based Therapeutic Strategies. *Biochimica et Biophysica Acta (BBA)–Molecular Basis of Disease*.

[39] Murata D., Arai K., Iijima M., Sesaki H. (2020). Mitochondrial Division, Fusion and Degradation. *The Journal of Biochemistry*.

[40] Whitley B. N., Engelhart E. A., Hoppins S. (2019). Mitochondrial Dynamics and Their Potential as a Therapeutic Target. *Mitochondrion*.

[41] Roy M., Reddy P. H., Iijima M., Sesaki H. (2015). Mitochondrial Division and Fusion in Metabolism. *Current Opinion in Cell Biology*.

[42] Endale H. T., Tesfaye W., Mengstie T. A. (2023). ROS Induced Lipid Peroxidation and Their Role in Ferroptosis. *Frontiers in Cell and Developmental Biology*.

[43] Shadel G. S., Horvath T. L. (2015). Mitochondrial ROS Signaling in Organismal Homeostasis. *Cell*.

[44] Deng J., Walther A. (2020). ATP-Responsive and ATP-Fueled Self-Assembling Systems and Materials. *Advanced Materials*.

[45] He C., Zhang X. L., Zhang Q., Ge L. Y., Ding W. X. (2021). Adiponectin Ameliorated Pancreatic Islet Injury Induced by Chronic Intermittent Hypoxia Through Inhibiting the Imbalance in Mitochondrial Fusion and Division. *Chinese Medical Sciences Journal*.

[46] Gallo M., Sapio L., Spina A., Naviglio D., Calogero A., Naviglio S. (2015). Lactic Dehydrogenase and Cancer: An Overview. *Frontiers in Bioscience*.

[47] Chen X., Yang T., Zhou Y., Mei Z., Zhang W. (2024). Astragaloside IV Combined With Ligustrazine Ameliorates Abnormal Mitochondrial Dynamics via Drp1 SUMO/deSUMOylation in Cerebral Ischemia-Reperfusion Injury. *CNS Neuroscience & Therapeutics*.

[48] Gao Z., Zhang C., Peng F. (2022). Hypoxic Mesenchymal Stem Cell-Derived Extracellular Vesicles Ameliorate Renal Fibrosis After Ischemia-Reperfusion Injure by Restoring CPT1A Mediated Fatty Acid Oxidation. *Stem Cell Research & Therapy*.

[49] Lu G., Li H. X., Song Z. W. (2024). Combination of Bone Marrow Mesenchymal Stem Cells and Moxibustion Restores Cyclophosphamide-Induced Premature Ovarian Insufficiency by Improving Mitochondrial Function and Regulating Mitophagy. *Stem Cell Research & Therapy*.

[50] Lossi L., Castagna C., Merighi A. (2018). Caspase-3 Mediated Cell Death in the Normal Development of the Mammalian Cerebellum. *International Journal of Molecular Sciences*.

[51] Hosseini A., Pourheidar E., Rajabian A., Asadpour E., Hosseinzadeh H., Sadeghnia H. R. (2023). Linalool Attenuated Ischemic Injury in PC12 Cells Through Inhibition of Caspase-3 and Caspase-9 During Apoptosis. *Food Science & Nutrition*.

[52] Xie H., Song L., Katz S. (2022). Electron Transfer Between Cytochrome c and Microsomal Monooxygenase Generates Reactive Oxygen Species That Accelerates Apoptosis. *Redox Biology*.

[53] Nolden K. A., Harwig M. C., Hill R. B. (2023). Human Fis1 Directly Interacts With Drp1 in an Evolutionarily Conserved Manner to Promote Mitochondrial Fission. *Journal of Biological Chemistry*.

[54] Song Z., Xia Y., Shi L. (2024). Inhibition of Drp1- Fis1 Interaction Alleviates Aberrant Mitochondrial Fragmentation and Acute Kidney Injury. *Cellular & Molecular Biology Letters*.

[55] Gao S., Hu J. (2021). Mitochondrial Fusion: The Machineries In and Out. *Trends in Cell Biology*.

[56] Li C., Liu Q., Chang Q. (2023). Role of Mitochondrial Fusion Proteins MFN2 and OPA1 on Lung Cellular Senescence in Chronic Obstructive Pulmonary Disease. *Respiratory Research*.

[57] Quiles J. M., Gustafsson Å. B. (2022). The Role of Mitochondrial Fission in Cardiovascular Health and Disease. *Nature Reviews Cardiology*.

[58] Chang X., Niu S., Shang M. (2023). ROS-Drp1-Mediated Mitochondria Fission Contributes to Hippocampal HT22 Cell Apoptosis Induced by Silver Nanoparticles. *Redox Biology*.

[59] Wang Y., Lu M., Xiong L. (2020). Drp1-Mediated Mitochondrial Fission Promotes Renal Fibroblast Activation and Fibrogenesis. *Cell Death & Disease*.

